# Coagulation parameters correlate to venous thromboembolism occurrence during the perioperative period in patients with spinal fractures

**DOI:** 10.1186/s13018-023-04407-y

**Published:** 2023-12-06

**Authors:** Yong Jiao, Xiaohong Mu

**Affiliations:** Department of Orthopedics, Dongzhimen Hospital, Beijing University of Traditional Chinese Medicine, No. 5 Haihai Warehouse, Dongzhimen, Beijing, 100000 China

**Keywords:** Venous thromboembolism, Spinal injury surgery, Coagulation, ROC, OR

## Abstract

**Background:**

Venous thromboembolism (VTE) is one of the leading causes of mortality in hospitalized patients. However, whether the coagulation-related parameters of the hospitalized patients could be used to predict the occurrence of VTE in patients with spinal injury surgery remained unclear.

**Method:**

The patients with spinal fractures who met the inclusion and exclusion criteria were enrolled to be analyzed using a retrospective analysis approach. The association of risk factors of enrolled patients and operations to VTE occurrence were analyzed. The activated partial thromboplastin time, prothrombin time, thrombin time, D-dimer (D-D), fibrinogen (FIB) and fibrinogen degradation products (FDP) were detected. ROC and HR analysis were applied to evaluate the correlation of coagulation-related parameters and other parameters to VTE occurrence.

**Result:**

The indicators of D-D, FIB and FDP were significantly elevated in VTE patients compared to non-VTE patients. The multivariate analysis of OR showed that six risk factors, including age ≥ 60, spinal cord injury, postoperative bedtime over 5 days, plasma D-dimer ≥ 0.54 mg/L, plasma fibrinogen ≥ 3.75 g/L and plasma FDP ≥ 5.19 mg/L, were positively correlated to VTE.

**Conclusion:**

The six risk factors, including D-D, FIB, FDP, age ≥ 60, spinal cord injury, and postoperative bedtime over 5 days, could be used to predict the occurrence of VTE.

## Introduction

Presently, venous thromboembolism (VTE) is one of the leading causes of mortality in hospitalized patients, accounting for 10% of all hospitalized patient deaths [1, 2]. VTE is a chronic disease with approximately 30% of VTE patients experiencing recurrence within 10 years [3]. It includes two types with deep vein thrombosis (DVT) and pulmonary thromboembolism (PE) [4, 5]. About 50% of untreated DVT cases are complicated by PE. Contrarily, 50–80% of all untreated PE cases are related to DVT [6, 7]. Therefore, VTE attracted more attention from surgeons and increasing studies reported on the risk factors of VTE.

Risk factors for VTE comprised three parts: factors that promote venous stasis, factors that promote blood hypercoagulability and factors causing endothelial injury or inflammation. These three broad categories are the main content of thrombosis theory, which was proposed by Virchow and endorsed by other researchers until now [8]. VTE is mainly composed of fibrin chains, red blood cells and a few platelets, which occur in the condition of a low-flow environment. The risk of VTE occurrence increases dramatically in clinical situations involving the three elements of Virchow. To identify individuals at risk of VTE who would benefit from thromboprophylaxis, a thorough understanding of the risk factors for VTE is crucial. Individual patients' risk of VTE is determined by intrinsic patient-specific characteristics such as genetic risk factors, age, or body mass index, as well as acquired risk owing to a particular context or event such as hospitalization, surgery, or cancer [9].

The type of surgery is a critical factor that results in the occurrence of VTE in patients. High-risk surgical operation includes orthopedic lower limb large joint surgery, thoracic, abdominal or pelvic malignant tumor surgery, neurosurgery surgery, kidney transplantation and cardiovascular surgery, all of these surgical operations could cause VTE [10]. In spinal surgery, the occurrence of VTE has specific characteristics. Firstly, the posterior approach to spine surgery adopts a prone position with a long surgical time, resulting in prolonged abdominal compression, which hinders venous reflux and promotes thrombosis. Secondly, intraoperative stimulation of the autonomic nerve leads to vasoconstriction, consequential blockage of venous return and slowing of blood flow. Thirdly, the operation may stretch, stimulate and crush the blood vessels, leading to damage of the blood vessel wall and thrombosis due to numerous great vessels in the operation area of the anterior approach. Fourth, the activation of the coagulation system caused by the trauma after surgery, blood concentration and hypercoagulable state of the patient due to blood loss, consequentially caused thrombosis.

With the growing awareness of perioperative venous thrombosis among domestic researchers, surgeons have recently focused on the thrombosis of these patients with spinal trauma. However, some VTE patients had uncommon clinical symptoms, leading the patient with lower limb DVT to overlook subjective discomfort. As a result, early clinical screening and surveillance are extremely crucial for VTE occurrence. Several studies have shown that preventing VTE is critical for improving the prognosis of postoperative patients. Early intervention of high-risk factors for VTE could effectively reduce the incidence of postoperative VTE, and its significance is greater than anticoagulation and thrombolysis treatment after VTE occurs. Therefore, exploring the risk factors for VTE after spinal surgery and constructing a risk prediction model for VTE could help surgeons effectively evaluate the risk of VTE, screen high-risk patients for VTE, take timely and effective preventive measures and consequently reduce the incidence of VTE in patients with spinal surgery.

There are currently multiple VTE risk scores available internationally to assess the VTE risk for different patients, including the Caprini score, Padua score, Wells score, Geneva score, and Simplified Geneva score. However, there is no suitable score tool for the prediction of VTE incidence in patients with spinal fractures. Coagulation-related parameters were one of the risk factors for VTE, however, whether the coagulation-related parameter could be used for the establishment of the risk prediction model for VTE patients with spinal fractures remained unknown. Taken together, the purpose of this study is to investigate the correlation between coagulation-related parameters at admission and perioperative VTE in patients with spinal traumatic fractures. This study adopts a retrospective analysis method to analyze the incidence of VTE during the perioperative period in 488 patients with spinal trauma fractures in our hospital over the past five years.

## Material and methods

### The inclusion and exclusion criteria for enrolled patients

The study was approved by Dongzhimen Hospital, Beijing University of Traditional Chinese Medicine, and informed written consent was derived from the participants. All the enrolled patients were included who met the following criteria: spinal trauma fracture requires spinal surgery for admission; age more than 18 years old; postoperative hospitalization time more than 72 h. If the patients who met the following criteria would be excluded from the analysis: merge fractures of other parts; non-traumatic fractures; with history of thrombosis; concomitant blood system diseases or long-term use of anticoagulants; preoperative VTE; missing or incomplete medical record information; patients with malignant tumors.

### The identification of VTE patients

All patients were reexamined within 7 days after the operation. Ultrasound examination of lower limb blood vessels was conducted when the patient has the possibility of DVT with clinical manifestations, such as lower limb swelling, pain, and skin cyanosis. CT pulmonary angiography should be performed immediately when the patient was suspected to be acute PE with sudden chest pain, dyspnea, hemoptysis and other symptoms. The patients were divided into a VTE group and a non-VTE group according to the occurrence of VTE during the perioperative period.

### The detection of coagulation-related parameters

The activated partial thromboplastin time (APTT), prothrombin time (PT), thrombin time (TT), D-dimer (D-D), fibrinogen (FIB) and fibrinogen degradation products (FDP) was detected according to the instruction of the manufacturer with an automated coagulation analyzer (CS-5100, Sysmex Corporation, RJDK, Japan).

### The receiver operating characteristics (ROC) and ORs analysis

The ROC analysis for D-D, FIB and FDP was conducted in SPSS software. The Youden index was determined by the value of sensitivity plus specificity, minus 1. The Joint model of prediction was determined according to the following formula: Logit (Joint model) = 4.344 * Plasma D-dimer + 1.311 * Plasma fibrinogen + 0.691 * Plasma FDP.

The VTE and NVTE groups of an enrolled patient with spinal trauma fractures were further divided into two subgroups according to age of more than 60 years old, spinal cord injury, or operation duration of more than 3 h, or postoperative bedtime of more than 5 days, or D-D more than 0.54 mg/L, or FIB more than 3.75 g/L, or FDP more than 5.19 mg/L. The patients with VTE occurrence in two different subgroups were recorded as 1, otherwise as 0. Then, the OR values for VTE occurrence between two subgroups from different risk factors were calculated in SPSS software.

### Statistical analysis

All statistical analysis was performed using SPSS software, version 26.0. All *p* values were determined by Fisher's exact test or Chi-square test, Mann–Whitney test or unpaired t-test with Welch's correction. *P* with < 0.05 indicated the significance of the test.

## Results

### The characterization of enrolled patients

In this study, 73 out of 488 eligible patients with spinal fractures experienced VTE during the perioperative period, which comprise 25 cases of DVT in the left lower limb, 27 cases of DVT in the right lower limb, and 21 cases of DVT in the double lower limb. One case was PE, and the PE patient was complicated with DVT in the lower limb. Among the 73 VTE patients, 9 occurred before surgery and 64 occurred after surgery. All the VTE patients did not receive tranexamic acid or similar drugs in their therapy. 415 cases are non-VTE (NVTE) patients.

As shown in Table 1, the percentage of patients with VTE whose age ≥ 60 was higher than patients whose age < 60, which indicated that the patients with spinal trauma fractures whose age was over 60 had a higher possibility to occur VTE. Moreover, the percentage of patients with spinal fractures complicated with spinal cord injury occurring VTE was significantly higher than that of patients with spinal fractures who are not complicated with spinal cord injury. This finding implied that the spinal cord injury promoted the occurrence of VTE in patients with spinal fractures. Additionally, the percentage of patients with spinal fractures occurring VTE increased dramatically when the operation duration was over 3 h and postoperative bed time was over 5 days, which revealed that these two risk factors including operation duration and postoperative bed time also significantly affected the occurrence of VTE. What’s more, the gender, fracture site, body mass, diabetes mellitus, hypertension, hyperlipidemia and operation history did not influence the VTE occurrence as shown in Table 1.

**Table 1:** Demographic and clinical characteristics of patients with spinal fractures underwent venous thromboembolism or not during perioperative period

Clinical factors	NVTE (*n* = 415)	VTE (*n* = 73)	*p* Value
Age (year)			
< 60	306 (73.7%)	41 (56.2%)	0.003
≥ 60	109 (26.3%)	32 (43.8%)	
Gender			
Male	226 (54.5%)	45 (61.6%)	0.307
Female	189 (45.5%)	28 (38.4%)	
Fracture site			
Cervical vertebra	69 (16.6%)	10 (13.7%)	0.737
Thoracic vertebra	103 (24.8%)	16 (21.9%)	
Lumbar vertebra	187 (45.1%)	38 (52.1%)	
Multi-segmental spine	56 (13.5%)	9 (12.3%)	
Body mass index > 28			
Yes	76 (18.3%)	17 (23.3%)	0.333
No	339 (81.7%)	56 (76.7%)	
Spinal cord injury			
Yes	98 (23.6%)	31 (42.5%)	0.001
No	317 (76.4%)	42 (57.5%)	
Diabetes mellitus			
Yes	49 (11.8%)	13 (17.8%)	0.181
No	366 (88.2%)	60 (82.2%)	
Hypertension			
Yes	83 (20%)	19 (26%)	0.274
No	332 (80%)	54 (74%)	
Hyperlipidemia			
Yes	74 (17.8%)	20 (27.4%)	0.075
No	341 (82.2%)	53 (72.6%)	
Operation history			
Yes	115 (27.7%)	26 (35.6%)	0.207
No	300 (72.3%)	47 (64.4%)	
Operation duration (hour)			
< 2	148 (35.7%)	12 (16.4%)	< 0.001
2–3	196 (47.2%)	37 (50.7%)	
> 3	71 (17.1%)	24 (32.9%)	
Postoperative bed time (day)			
< 3	109 (26.3%)	9 (12.3%)	0.004
3–5	185 (44.6%)	30 (41.1%)	
> 5	121 (29.1%)	34 (46.6%)	

The data are presented as n (percentage). The comparisons of data between the two group were done by Fisher’s exact test or Chi-square test

### The correlation of coagulation-related parameters and VTE occurrence

**Fig. 1:**
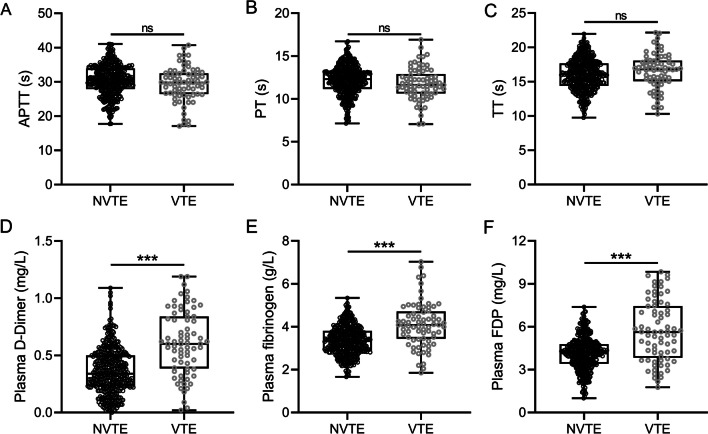
Coagulation-related parameters detection in VTE and NVTE groups. **A**–**F** Comparisons of activated partial thromboplastin time (APTT) (**A**), prothrombin time (PT) (**B**), thrombin time (TT) (**C**), plasma D-Dimer (**D**), plasma fibrinogen (FIB) (**E**) and fibrinogen degradation products (FDP) (**F**) at admission in patients with spinal fractures underwent venous thromboembolism (VTE, *n* = 73) or not (NVTE, *n* = 415) during perioperative period. Data were shown with a Box plot. ns, no significance; ***, *p* < 0.001 from the Mann–Whitney test or unpaired *t*-test with Welch’s correction

APTT is a commonly used coagulation assay and reflected the coagulation function of coagulation factors in the body [11]. The value of APTT between NVTE and VTE had no significant difference, which indicated that APTT did not affect the VTE occurrence as shown in Fig. 1A. PT is an index reflecting the activity of coagulation factors I, II, V, VII and X in plasma [12]. The analysis of PT presented that there is no difference between NVTE and VTE groups as shown in Fig. 1B. Similarly, there was no significant difference in TT (Fig. 1C), which reflects the conversion of fibrinogen to fibrin after the addition of a thrombin reagent [13]. Interestingly, plasma D-dimer is a biomarker used as an exclusion criterion of VTE disease [14], the level of it was elevated in the patients with VTE compared with patients without VTE as shown in Fig. 1D. Meanwhile, the level of FIB and FDP also increased dramatically in patients with VTE compared with patients without VTE (Fig. 1E, F). Taken together, the time-related parameters were not correlated to the occurrence of VTE; nevertheless, the parameters including D-D, FIB and FDP were closely associated with VTE occurrence in patients.

### The prediction model of VTE occurrence by coagulation-related parameters

**Fig. 2:**
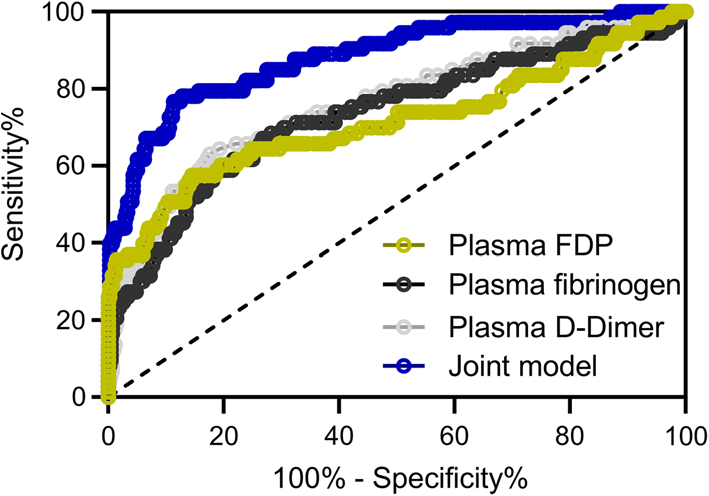
ROC analysis of plasma D-Dimer, plasma fibrinogen, fibrinogen degradation products (FDP) at admission and their joint test model for the prediction of venous thromboembolism occurrence during the perioperative period in patients with spinal fractures

It was well known that three coagulation-related parameters, D-D, FIB and FDP, were closely correlated to VTE occurrence, however, whether these parameters could be used as a predictor for the VTE occurrence needs further exploration. The ROC analysis of these three parameters revealed that the AUC of those was over 0.7, which implied that the prediction of VTE occurrence using these three parameters had some accuracy (Fig. 2, Table 2). Importantly, the joint model which integrated three parameters predicted the VTE occurrence with 0.88 AUG more accurately (Fig. 2, Table 2). Meanwhile, the sensitivity and specificity of the joint model also greatly improved compared to that of the single factor prediction model (Table 2). Collectively, it was concluded that the joint model could be the predictor for VTE occurrence.

**Table 2:** Predictive values in ROC analysis

	AUC	95% CI	Cut off	Sensitivity (%)	Specificity (%)	Youden index
Plasma D-Dimer	0.76	0.69–0.83	0.54	63.01	82.41	0.45
Plasma fibrinogen	0.73	0.66–0.80	3.75	67.12	73.25	0.40
Plasma FDP	0.71	0.64–0.79	5.19	57.53	85.54	0.43
Joint model	0.88	0.84–0.93	–	76.71	88.67	0.65

*CI* confidence interval, *FDP* fibrinogen degradation products

^&^Logit (Joint model) = 4.344 * Plasma D-Dimer + 1.311 * Plasma fibrinogen + 0.691 * Plasma FDP

### Multivariate logistic analysis for VTE occurrence

The above findings unveiled that age, spinal cord injury, operation duration, postoperative bed time and coagulation-related parameters were correlated to VTE occurrence. Afterwards, the ORs of all those factors in VTE occurrence were analyzed using multivariate analysis. The result showed that all ORs for those risk factors were over 1 significantly except for operation duration (Table 3). These findings demonstrated that the patients, whose age were over 60 years or with spinal cord injury, were more likely to develop VTE. Moreover, the patients whose postoperative bed time is more than 5 days had a higher possibility to occur VTE. Importantly, the patients with D-D over 0.54 mg/L, or FIB over 3.75 g/L, or FDP over 5.19 mg/mL were also easier to have VTE occurrence, which was indicated by the corresponding ORs with 2.72, 1.89 and 1.49 (Table 3). The multivariate analysis revealed that six risk factors could be used as indicators for VTE occurrence with ORs more than 1 significantly.

**Table 3:** Multivariate logistic analysis for venous thromboembolism occurrence during perioperative period in patients with spinal fractures

	OR	95% CI	*p* Value
Age more than 60 years old	2.13	1.38–4.82	0.002
With spinal cord injury	1.64	1.03–3.68	0.026
Operation duration more than 3 h	1.75	0.97–2.89	0.091
Postoperative bed time more than 5 days	2.36	1.08–5.37	0.014
Plasma D-Dimer more than 0.54 mg/L	2.72	1.21–6.09	0.007
Plasma fibrinogen more than 3.75 g/L	1.89	1.17–3.65	0.009
Plasma FDP more than 5.19 mg/L	1.49	1.05–4.43	0.018

*OR* odds ratio, *CI* confidence interval, *FDP* fibrinogen degradation products

## Discussion

VTE has become the third most prevalent vascular disease following acute coronary syndrome and stroke; it is also a common complication following surgery. However, the risk assessment model for VTE in patients with spinal fractures is scarce. Our analysis revealed that six factors, including age, spinal cord injury, Postoperative bed time, D-DI, FIB and FDP, were correlated to the occurrence of VTE, respectively. Importantly, the AUG of the joint model for VTE risk assessment was 0.88 with higher sensitivity and specificity compared to the D-D, FIB and FDP, respectively.

It was reported that various risk factors contributed to the occurrence of VTE in patients with spinal fractures, which could be divided into non-genetic and genetic factors. In elderly patients, hardening of blood vessels, a decrease in elasticity of blood vessel wall and venous valve function led to slow of blood flow. In addition, hypertension, diabetes and other basic diseases, accompanied by functional or organic changes of multiple organs, are likely to lead to hypercoagulability of the body's blood, consequently increasing the risk of VTE [15, 16]. Previous studies showed that the incidence of lower limb DVT increases by 0.5–0.6 percent when age increases from 45 to 89 years old [17]. Masuda et al. [18] found that thrombosis incidence with over 70 years old significantly increased compared with patients with lower than 50 years old in patients after spinal surgery. Our findings also unveiled that the VTE incidence was higher in patients over 60 years old, which was in line with the previous findings. Although some studies suggested that the occurrence of VTE is not related to age [19], the Chinese thrombosis prevention recommendations state unequivocally that the older one is the greater the risk of lower limb DVT.

Patients with spinal fractures, often complicated with spinal cord injury, are more prone to VTE, compared to patients without spinal cord injury. In previous investigations, spinal cord damage is a significant risk factor for VTE among patients obtaining spinal fracture surgery. Moreover, spinal cord damage is frequently associated with traumatic events, neurosurgical operations, and protracted immobility, all of which are high-risk factors for VTE [20]. Our study revealed that the percentage of VTE was 24% in patients with spinal fractures complicated by spinal cord injury, which was consistent with previous research [21]. Besides, it was reported that longer operative duration was associated with an increased risk of VTE [22]. In our study, the operation duration was significantly correlated to the incidence of VTE, however, the OR of operation duration for VTE occurrence in the multivariate logistic analysis showed that the patients with longer operation duration over 3 h did not have higher risk for VTE. Additionally, slow blood flow caused by postoperative bed time increased the incidence of lower limb DVT. Our study showed that patients with postoperative bed time of more than 5 days had a higher incidence of VTE.

The non-genetic factors, including age, spinal cord injury, operative duration and postoperative bed time, were proved to be associated with VTE occurrence. Meanwhile, APTT, PT and TT also attributed to non-genetic factors for VTE occurrence. Interestingly, other studies presented that APTT and PT had a significant difference between NVTE and VTE groups, nevertheless, TT did not have a significant difference between NVTE and VTE groups [23]. Our findings revealed that all of these three parameters, including APTT, PT and TT, did not have differences among NVTE and VTE groups, which was not consistent with other studies. The possible reason might be due to the differences in the number of patients included in the analysis. Another possible reason was that the exclusion criteria were different from other studies. Therefore, whether these three parameters were closely correlated to the VTE occurrence needs more patients for analysis.

Except for the non-genetic factors, the genetic factors associated with the coagulation were also involved in the VTE occurrence. When fibrin is degraded by plasmin, D-dimer is produced, which is a product of early fiber degradation in thrombosis. The elevation of DD has been widely confirmed as a risk factor for VTE occurrence [24, 25]. Our finding also unveiled that DD was significantly elevated in the VTE group compared to the NVTE group, which indicated that DD could be an independent risk factor for VTE incidence. Meanwhile, it was also demonstrated that high FIB was associated with susceptibility to thrombosis in patients with posttraumatic deep vein thrombosis [26]. FIB level was significantly increased in the patient with VTE compared to the patient without VTE in our study. Additionally, FDP is also a product of fibrinogen degradation. Wu et al. [27] analyzed 569 patients with femoral and pelvic fractures, indicating that a high level of FDP is a high-risk factor for DVT in the perioperative period. It was also proved that FDP was dramatically increased in patients with VTE in our study. Taken together, DD, FIB and FDP could be the risk factor for VTE incidence in patients with spinal fractures separately. Importantly, the combination of these three factors was more effective to predict the incidence of VTE in patients with spinal fractures.

Although our study revealed the non-genetic factors and genetic factors were closely correlated to VTE incidence through chi-square test and OR analysis, the number of included patients was limited so that the bias could not be excluded from the analysis. More patients would be included in the risk assessment of VTE incidence.

## Conclusion

This study demonstrated that six factors were related to VTE incidence in patients with spinal fractures, where the combination of DD, FIB and FDP could be as the joint model to predict the occurrence of VTE in patients with spinal fractures effectively.

## Data Availability

The raw data supporting the conclusions of this article will be made available by the authors.

## Abbreviations

OR: Odds ratio
CI: Confidence intervals
FDP: Fibrinogen degradation products
APTT: Activated partial thromboplastin time
PT: Prothrombin time
TT: Thrombin time
DD: Plasma D-dimer (D)
FIB: Plasma fibrinogen
FDP: Fibrinogen degradation products
